# Impact of universal varicella vaccination on 1-year-olds in Uruguay: 1997–2005

**DOI:** 10.1136/adc.2007.126243

**Published:** 2008-05-02

**Authors:** J Quian, R Rüttimann, C Romero, P Dall’Orso, A Cerisola, T Breuer, M Greenberg, T Verstraeten

**Affiliations:** 1 Pediatric Department, Republic University, Montevideo, Uruguay; 2 GlaxoSmithKline Biologicals, Buenos Aires, Argentina; 3 Pediatric Clinic, Republic University, Montevideo, Uruguay; 4 GlaxoSmithKline Biologicals, Rixensart, Belgium

## Abstract

**Objective:**

Varicella vaccination was introduced at the end of 1999 into the Uruguayan immunisation schedule for children aged 12 months. *Varilrix* (Oka strain; GlaxoSmithKline Biologicals) has been the only vaccine used since then and coverage has been estimated to exceed 90% since the start of the universal varicella vaccination programme. We assessed the impact of the Uruguayan varicella vaccination programme during 2005, 6 years after its introduction.

**Methods:**

Information on hospitalisations was collected from the main paediatric referral hospital and information on medical consultations for varicella was collected from two private health insurance systems in Montevideo. The proportion of hospitalisations due to varicella and the proportion of ambulatory visits for varicella since the introduction of the vaccine were compared between 1999 and 2005 and 1997 and 1999 in the following age groups: <1 year, 1–4 years, 5–9 years and 10–14 years.

**Results:**

By 2005, the proportion of hospitalisations due to varicella among children, was reduced by 81% overall and by 63%, 94%, 73% and 62% in the <1, 1–4, 5–9 and 10–14 years age groups, respectively. The incidence of ambulatory visits for varicella among children was reduced by 87% overall and by 80%, 97%, 81% and 65% in the <1, 1–4, 5–9 and 10–14 years age groups, respectively.

**Conclusions:**

The burden of varicella has decreased substantially in Uruguayan children since the introduction of the varicella vaccination, including those groups outside the recommended vaccination age. It is expected to decrease further as more cohorts of children are vaccinated and herd immunity increases.

Although varicella is usually considered a mild childhood disease, it carries a considerable public health burden as it affects nearly all children before the age of 10 and is not devoid of complications. Before implementation of routine varicella vaccination in the USA, varicella caused an estimated 11 000 hospitalisations and 100 deaths every year (or approximately 2.6 deaths per 100 000 cases), of which approximately 43 deaths occurred in mostly healthy children <15 years of age.^1^ ^2^

Numerous clinical trials have shown that varicella vaccines derived from the Oka strain, isolated in Japan in the early 70s, are safe, immunogenic and protective against varicella in healthy children and adults.^3^ ^4^ These results have been confirmed by post-licensure surveillance studies in the USA^5^^–^7 and Israel.^8^

The two most widely used varicella vaccines are *Varivax* from Merck & Co. and *Varilrix* from GlaxoSmithKline Biologicals. In 1995, the USA became the first country to include varicella into the routine immunisation calendar using a frozen vaccine formulation (*Varivax*). From 1995 to 2000, varicella cases in the USA declined by 71% to 84%, depending on the region, with the largest decrease observed in children aged 1–4 years. Despite irregular acceptance and overall vaccination coverage below 75%, 10 years of routine immunisation reduced varicella-related deaths from approximately 100, to 26 per year, with the highest reduction (92%) also observed in children aged 1–4 years.^9^ Hospitalisations similarly declined from a range of 2.7–4.2 per 100 000 population from 1995 to 1998, to 0.6 and 1.5 per 100 000 population in 1999 and 2000, respectively.^10^ Routine varicella vaccination of young children is now also recommended in Canada (*Varilrix* and *Varivax*),^11^ Germany (*Varilrix* and *Varivax*),^12^ Australia (*Varilrix* and *Varivax*),^13^ Qatar (*Varilrix*),^14^ Taiwan (*Varivax*)^15^ and South Korea (*Varilrix* and *Varivax*),^16^ and is under consideration in other European countries.^17^

Among developing middle-income countries, Uruguay is characterised by a modern and efficacious immunisation policy. At the end of 1999, universal vaccination against varicella was introduced among children 12 months of age.^18^ No catch-up vaccination took place. The varicella vaccine used in Uruguay, *Varilrix* is, like the US-licensed vaccine *Varivax*, derived from the Oka strain. *Varilrix* differs from the US formulation of *Varivax* in its temperature stability, whereby *Varilrix* does not need to be stored in the freezer.^4^

Vaccines that are part of the national recommended immunisation schedule in Uruguay are offered free of charge. *Varilrix* is administered concomitantly with the measles, mumps and rubella (MMR) vaccine, and vaccination is mandatory before kindergarten and school entry. According to the Uruguayan Ministry of Public Health this has resulted in a varicella vaccination coverage of 96%, 96%, 93%, 96%, 94% and 88%, respectively, for children born in 1999, 2000, 2001, 2002, 2003 and 2004,^19^ with similar coverage continuing during 2005 (J Quian, personal communication, 2006). The aim of this study was to assess the impact of the varicella universal vaccination programme in Uruguay on health service utilisation for varicella in children, 6 years after the start of the programme.

## MATERIALS AND METHODS

We conducted a study of utilisation of health services for varicella infection in the 3 years before and 6 years after introduction of varicella vaccination (January 1997–December 2005). Data collection was retrospective between 1997 and 2003 and prospective from 2004.

### Vaccine

One dose of *Varilrix* (0.5 ml) contains 10^3.3^ plaque forming units of live attenuated varicella-zoster (Oka strain) virus, propagated in MRC5 human diploid cells. *Varilrix* is stored by refrigeration at +2°C to +8°C and is not affected by freezing (refrigeration-stable formulation registered in 1994).

### Study population

The study was conducted in Montevideo, the capital of Uruguay. According to the latest census in 1996, Montevideo has a total population of 1 344 839 among whom 294 831 are <15 years old with the following age distribution: 20 081 aged less than 1 year (representing 1.5% of the total population); 77 398 aged between 1 and 4 years (5.8%); 100 028 aged between 5 and 9 years (7.4%); and 97 324 aged between 10 and 14 years (7.2%).^20^

### Health surveillance systems

Both public and private health surveillance systems are in place in Uruguay. We obtained data from both systems. The public system serves a population that is generally more disadvantaged, relying on the Ministry of Public Health for healthcare. The main paediatric referral centre in this sector is the Centro Hospitalario Pereira Rossell (CHPR). This is the only paediatric referral hospital in Uruguay and admits cases of varying severity, including children with complex and severe illness. Data on hospitalisations related to varicella were obtained from the ward registers and automated database of this hospital. All cases were recorded, regardless of whether or not varicella was the presenting condition.

The private health system consists of several organisations that service a substantial proportion of the population (25% of children <15 years in the city of Montevideo). These organisations provide ambulatory medical services (home care, consultations at polyclinics and hospital transfer in case of emergency). Two private insurance organisations were involved in the present study: Servicio de Urgencia Asistencia y Traslado (SUAT) and Servicio de Emergencia Medico Movil (SEMM), respectively, serving an average of 63 000 and 19 000 children aged <15 years (approximately 10% of Uruguayan children in total). At SEMM, data were only available from 1998 onwards. Data on ambulatory visits for varicella were extracted from hand-written registers for the period before 1999 and from automated databases from 1999 onwards.

Age, gender and place of residence were collected for all reported varicella cases in the three institutions. For hospitalised cases the following data were also obtained: diagnosis at the time of admission, number of hospitalisation days, varicella immunisations, admission or not in the Intensive Care Unit (ICU), and final diagnosis and outcome for the patient.

### Statistical analysis

The CHPR does not cover a well-defined population and it was not possible to calculate or compare varicella hospitalisation rates. Instead, the proportion of admissions for varicella compared to the overall number of admissions was calculated. For ambulatory visits, incidence rates could be calculated and compared over time since membership of the private insurance systems providing the ambulatory varicella numbers was exactly known.

**Figure 1:**
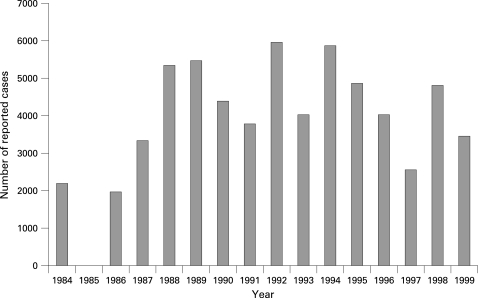
Yearly number of varicella cases reported to the Uruguayan MOH, 1983–1999 (Ministerio de Salud Pública, Departamento de Vigilancia Epidemiológica). (1985: no data). MOH, Ministry of Health.

Surveillance data from the Ministry of Health show varicella notifications prior to the introduction of routine varicella vaccination (fig 1). Surveillance was similar for all years except for 1985, where no data were available because the programme was new and recently implemented. To estimate the burden of varicella disease in the pre-vaccination period, we calculated the proportions and incidence rates for the entire period 1997–1999 to compensate for these cycles. As data were unavailable for 1997 at SEMM we used the 1998–1999 average in this system.

Fisher exact tests were used to test the decrease between the rates before and since the varicella vaccination programme. Statistical analyses used SAS V8.2 (SAS institute, Cary, North Carolina).

## RESULTS

### Hospitalisations

The total number of hospitalisations in the CHPR increased from 8130 in 1997 to 15 265 in 2005. The number of varicella-related hospitalisations varied from a maximum of 101 in 1998 to a minimum of 28 in 2005. The mean duration of varicella-related hospitalisation was 4 days in 1997–1999 and 3, 4, 5, 3.5, 4.8 and 3.8 days in 2000, 2001, 2002, 2003, 2004 and 2005, respectively.

The proportion of hospitalisations due to varicella, reduced by 81% overall (p<0.001) and by 63% (p<0.001), 94% (p<0.001), 73% (p<0.001) and 62% (p = 0.044) by 2005 in the <1, 1–4, 5–9 and 10–14 years age groups, respectively, compared to the preceding years from 1997 to 1999 (table 1).

**Table 1:** All and varicella-related hospitalisations in the Centro Hospitalario Pereira Rossell (CHPR), Uruguay, between 1997 and 2005

Age (years)	1997	1998	1999	2000	2001	2002	2003	2004	2005	% Reduction (p value*)
<1										
Total (N)	3081	2904	2779	3238	3901	3676	3664	4073	4094	63% (p<0.001)
Varicella (n)	8	33	28	23	16	17	14	21	12	
Proportion (%)	0.26	1.14	1.01	0.71	0.41	0.46	0.38	0.52	0.29	
1–4										
Total (N)	2760	2709	2761	3439	4940	4676	3980	4645	4448	94% (p<0.001)
Varicella (n)	28	48	44	10	9	11	7	3	4	
Proportion (%)	1.01	1.77	1.59	0.29	0.18	0.24	0.18	0.06	0.09	
5–9										
Total (N)	1224	1308	1468	1933	2818	2935	2999	3477	3432	73% (p<0.001)
Varicella (n)	11	11	13	5	5	13	10	11	8	
Proportion (%)	0.90	0.84	0.89	0.26	0.18	0.44	0.33	0.32	0.23	
10–14										
Total (N)	1014	1085	1267	2325	3513	3510	2580	3136	3144	62% (p = 0.044)
Varicella (n)	1	9	4	4	2	4	4	7	5	
Proportion (%)	0.10	0.83	0.32	0.17	0.06	0.11	0.16	0.22	0.16	
Unknown age†	51	14	43	212	434	475	0	313	147	
Total										
Total (N)	8130	8020	8318	11 147	15 606	15 272	13 223	15 644	15 265	81% (p<0.001)
Varicella (n)	48	101	89	42	32	45	35	42	28	
Proportion (%)	0.59	1.26	1.07	0.38	0.21	0.29	0.26	0.27	0.18	
Characteristics of cases										
Male/female (n)	26/22	47/54	46/43	23/19	22/10	22/23	21/14	22/20	11/17	
Without underlying condition	42	88	80	40	29	41	33	34	26	
Immunosuppressed/malnourished‡	3	8	8	2	2	4	0	7	2	
Other	0	0	0	0	1	0	1	1	0	
Unknown	3	5	1	0	0	0	1			

Other, celiac disease, nephritic syndrome or diabetes.

*Fisher exact test, % reduction from 1997 to 1999 until 2005.

†There were no varicella cases in individuals of unknown age.

‡Immunosuppression due to leukaemia, disease requiring corticosteroid therapy or HIV.

N, total number of hospitalisations; n, number of varicella-related hospitalisations.

The majority of varicella cases had no underlying condition (table 1). There was no change in the proportion of hospitalised cases with underlying conditions over time. When present, immunosuppression and malnourishment were the most common preceding conditions. Since the start of the varicella vaccination campaign in 1999, only six hospitalisations took place in vaccinated children; in 2002, a child with nephrotic syndrome who presented 7 days after vaccination; in 2003, a child with skin super-infection; in 2004, a 4-year-old with cellulitis who had been vaccinated 2 years previously; in 2005, a 2½-year-old child receiving treatment for rhabdomyosarcoma who was vaccinated at 12 months of age; a child with febrile seizures aged 2 years and 5 months who was vaccinated 4 months previously; and another child of 5 years with pneumonia who had been vaccinated at 3 years of age.

### Outpatient consultations

The combined membership <15 years of age for SUAT and SEMM decreased from approximately 85 000 in 1998 to 65 000 in 2005. In the period 1997–1999, the incidence of medical visits for varicella per 1000 members in the two systems was 38.8 overall and 16.2, 64.8, 44.9 and 13.9 for the <1, 1–4, 5–9 and 10–14 years age groups, respectively. In 2005, these incidence rates were reduced by 87% overall (p<0.001) and by 80% (p<0.001), 97% (p<0.001), 81% (p<0.001), and 65% (p<0.001) for the same respective age groups (table 2).

**Table 2:** Number and distribution of varicella cases in children aged <15 years in Uruguayan private healthcare providers between 1997 and 2005

Age (years)	Servicio de Urgencia Asistencia y Traslado (SUAT)									Servicio de Emergencia Medico Movil (SEMM)							
	1997	1998	1999	2000	2001	2002	2003	2004	2005	1998	1999	2000	2001	2002	2003	2004	2005
<1																	
Cases	63	86	52	22	31	14	11	7	6	106	39	46	35	17	6	16	10
Members	911	994	1064	1317	1245	2181	5446	3336	2396	8743	9604	8835	4409	3414	3223	2475	2513
Cases/1000	69.2	86.5	48.9	16.7	24.9	6.4	2.0	2.1	2.5	12.1	4.1	5.2	7.9	5.0	1.9	6.5	4.0
1–4																	
Cases	723	890	454	217	166	89	44	9	18	1038	369	271	165	44	23	42	26
Members	3796	4227	4787	5191	5633	7202	7106	7809	7966	20 114	20 659	19 243	17 517	14 133	13 869	9963	11 483
Cases/1000	190.5	210.6	94.8	41.8	29.5	12.4	6.2	1.2	2.3	51.6	17.9	14.1	9.4	3.1	1.7	4.2	2.3
5–9																	
Cases	592	709	379	245	299	248	188	79	43	769	300	248	267	138	137	187	123
Members	4148	4698	5238	5690	6218	7660	8491	7556	5950	23 383	23 826	22 490	20 725	16 913	16 715	11 854	13 894
Cases/1000	142.7	150.9	72.4	43.1	48.1	32.4	22.1	10.5	7.2	32.9	12.6	11.0	12.9	8.2	8.2	15.8	8.9
10–14																	
Cases	131	182	99	83	87	73	54	37	30	211	102	74	79	35	53	78	72
Members	3607	4074	4710	5190	5599	6840	7512	6430	7671	18 992	20 608	20 334	19 696	16 440	13 069	13 262	13 173
Cases/1000	36.3	44.7	21.0	16.0	15.5	10.7	7.2	5.8	3.9	11.1	4.9	3.6	4.0	2.1	4.1	5.9	5.5
Total																	
Cases	1509	1867	984	567	583	424	297	132	97	2124	810	639	546	234	219	323	231
Members	12 462	13 993	15 799	17 388	18 695	23 883	28 555	25 131	23 983	71 232	74 697	70 902	62 347	50 900	46 876	37 554	41 064
Cases/1000	121.1	133.4	62.3	32.6	31.2	17.8	10.4	5.3	4.0	29.8	10.8	9.0	8.8	4.6	4.7	8.6	5.6

**Figure 2:**
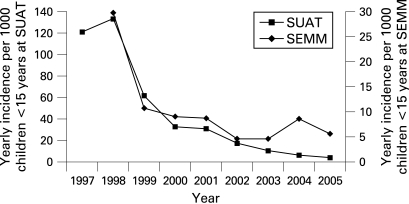
Incidence of varicella in children aged <15 years, Uruguay 1997–2005. SEMM, Servicio de Emergencia Medico Movil; SUAT, Servicio de Urgencia Asistencia y Traslado.

The evolution of the incidence of varicella in each system is illustrated in fig 2.

There were 358 varicella cases treated as outpatients among the three service providers during 2005, who were in the target age group (>1 year of age) and for whom clinical information, including vaccination status was available (tables 1 and 2). Of these, 118 had been previously vaccinated. Clinical varicella differed significantly in previously vaccinated and unvaccinated individuals: 78.0% of vaccinated subjects had ⩽50 lesions and none had >150 lesions compared with 32.9% and 23.8% of unvaccinated children, respectively (p<0.001). Fever was present in 39.8% of vaccinated subjected versus 77.1% of unvaccinated subjects (p<0.001) and was >38.5°C in 31.9% and 48.6% vaccinated and unvaccinated children, respectively (p = 0.003). Complications occurred in 6.8% of vaccinated children (cutaneous super-infection and vesicular haemorrhage) compared with 11.7% of unvaccinated children (cutaneous super-infection, bacterial super-infection, sepsis, scarlet fever and cervical adenopathy) (p>0.05). Hospitalisation occurred in 0.0% and 1.2% of vaccinated and unvaccinated children, respectively (p>0.05).

## DISCUSSION

The results of this retrospective study demonstrate significant reductions in the proportion of varicella hospitalisations in a large public paediatric hospital (up to 86% in the age group eligible for vaccination) compared to the pre-vaccination years 1997–1999. Furthermore, a substantial decrease was also observed in the ambulatory cases seen at two ambulatory healthcare providers (up to 88% in the age group eligible for vaccination). Clinical varicella, when it occurred in vaccinated subjects, was associated with significantly fewer and less severe symptoms and complications than disease in unvaccinated children.

The observed decrease in varicella incidence is neither linear, nor constant. The initial sharp decline in 1999 is likely to be due to cyclical increase and decrease of varicella rather than the vaccination campaign.^21^ However, the persistence of this decrease can be attributed to vaccination and it seems justified to assert that sustained reductions in case numbers and hospitalisations observed in the years following the onset of vaccination represent the true impact of the varicella vaccination programme. Without the varicella vaccination campaign, one would likely have seen another cyclical peak and a new increase after 2000. This is supported by data from Buenos Aires, an adjacent province in Argentina which has no varicella mass vaccination, where a doubling of cases was reported from 2001 to 2002.^22^ The slight increase in hospitalisations in 2002 and 2004 may represent the same cyclical phenomenon, but may have also been a result of the deteriorating economic climate in Uruguay that led to more hospitalisations for social reasons.

We observed a fourfold difference in the incidence rates between the two ambulatory healthcare providers. We do not have a clear explanation for this. We can only speculate that the thresholds for utilising the medical services seem to differ. However, as the trends are almost identical between the two systems (fig 2), we believe that the potential under-reporting in one system does not invalidate our findings.

Besides the substantial reduction in incidence in the 1–4-year-old group that were eligible for vaccination, large decreases were also observed in the other age groups. We interpret these decreases as indications of herd immunity already measurable in Uruguay. In the infant group, the reduction obtained by herd immunity was nearly as significant as the reduction observed in the vaccinated group. Similar observations were made in the USA.^9^

Uruguay has a relatively mild climate with average yearly temperatures of 12°C–25°C. Its varicella epidemiology should be expected to be similar to countries with mild climates in the Northern hemisphere, including parts of the USA. Our estimates of the incidence in the pre-vaccine period as well as the proportional reduction achieved by universal mass vaccination against varicella (UMVV) were comparable to those estimated in the USA, where Merck’s *Varivax* vaccine is the only vaccine in use.^10^ Table 3 summarises varicella epidemiology of both countries before and 5 to 6 years after introduction of UMVV.

**Table 3:** Impact of varicella universal vaccination in the USA (1995–2000)^10^ and Uruguay (1997–2005)

	Incidence before varicella vaccination (per 1000 population)		Reduction since varicella vaccination* (%)	
	USA	Uruguay	USA	Uruguay
<1 year	19.7	14.3	68–81	63–80
1–4 years	48.8	56.7	83–90	94–97
5–9 years	34.5	41.6	63–77	73–81
10–14 years	9.5	13.2	65–80	62–65

*Varicella vaccination started in the USA in 1995 and in Uruguay in 1999.

The economic crisis of 2001 had a clear impact on Uruguayan health services. We noted an increase in hospitalisations in the public hospital and a decrease in the membership of one of the two private healthcare services. In order to assess the potential impact of this situation on measured incidence rates, we calculated the average number of consultations per member of the ambulatory systems (SEMM and SUAT). This average did not decline during the study period (1.7 in 1998–1999 and 2.4 in 2002 when the combined membership was lowest), suggesting that the decline observed for varicella was not due to a reduced utilisation of the services.

Our study has some limitations. By its retrospective nature we could not confirm reported varicella cases by laboratory testing. It is possible that varicella cases occurring in the current high-coverage setting are milder than before, particularly among vaccinees, and are therefore more easily missed. This would have caused us to overestimate the impact of the varicella vaccination programme on the absolute number of varicella cases. We could not estimate hospitalisation incidence rates as the population that makes use of the CHPR is not well defined and is expected to vary with changing economic conditions. We believe that an economic factor, namely the severe economic crisis in Uruguay during 2001–2002, explains the increase in overall hospitalisations at this public hospital. This is supported by the decrease in overall hospitalisations in 2003, in parallel with slightly improving economic conditions and an increase in following years that also paralleled a fall in economic conditions. Public outpatient varicella attendances were not available and it is possible that outpatient attendance and hospitalisation rates may have differed within the public and private healthcare sectors. Finally, varicella vaccination started in 1999, thus we have not had opportunity to observe varicella breakthrough cases that may potentially occur many years after vaccination.

Two theoretical concerns have hindered the widespread acceptance of varicella vaccination beyond a handful of countries. The first concern is that vaccination against varicella might increase the overall morbidity related to the disease, due to an upward shift in the average age of infection from childhood to adulthood where risk of complication is greater.^23^ ^24^ Data from adolescents and young adults over a longer period of observation are required to confirm this observation. The second concern is that UMVV might increase the incidence of herpes zoster in older age groups, as it has been suggested that repeated exposure to varicella and the resulting natural boosting of immunity to the latent virus could reduce the risk of its reactivation.^25^ ^26^ We intend to test the hypothesised temporary increase of zoster by expanding the surveillance in Uruguay to include herpes zoster in children as well as adults, which began in 2004. In 2006 the USA adopted a two-dose varicella vaccination strategy, introducing a second varicella dose at 4–6 years of age with the specific aim of minimising primary vaccine failures and reducing the incidence of breakthrough disease.^27^ This strategy is also likely to inhibit potential upward age shifts in disease incidence and may have a positive impact on the incidence of zoster in later life.

This study assessed the impact of *Varilrix* when used under routine circumstances in a public health programme. We have shown that universal mass vaccination against varicella in Uruguay has highly beneficial effect in Uruguay by reducing both hospitalisations (by 81% overall) and outpatient visits (by 87% in children <15 years) for this disease. This beneficial effect is expected to increase further as more cohorts of children are vaccinated and herd protection increases. Unlike the USA, where varicella vaccination coverage has increased rather progressively, Uruguay serves as an example of the public health impact of universal varicella vaccination when the uptake is high from the very start of the programme.

**What is already known on this topic**

- Although safe and effective vaccines against varicella disease exist, universal mass vaccination against varicella (UMVV) is currently undertaken by very few countries.
- Vaccine effectiveness in preventing disease and death has been established by data from the USA where UMVV has been underway for a decade using a vaccine from a single manufacturer.
- Outside the USA there is a paucity of population effectiveness data in UMVV settings.

**What this study adds**

- This study provides vaccine effectiveness data following 6 years of UMVV in Uruguay, with more than 90% coverage since the start of the programme.
- The data specifically describe the effect of UMVV on hospitalisations and outpatient health service utilisation. The findings complement and add to those already published by US investigators.
- The vaccine administered in Uruguay is made by a different manufacturer to the US product and provides additional evidence that supports the effectiveness of Oka-strain vaccines in preventing disease and hospitalisations due to varicella.
